# A mixed-methods, exploratory, quasi-experimental evaluation of a radio drama intervention to prevent age-disparate transactional sex in Tanzania

**DOI:** 10.3389/frph.2022.1000853

**Published:** 2022-12-02

**Authors:** Marjorie Pichon, Daniel J Carter, Lottie Howard-Merrill, Revocatus Sono, Veronicah Gimunta, Oscar Rutenge, Yandé Thiaw, Kirsten Stoebenau, Nancy Perrin, Ana Maria Buller

**Affiliations:** ^1^ Gender Violence & Health Centre, Department of Global Health and Development, London School of Hygiene & Tropical Medicine, London, United Kingdom; ^2^ Department of Education, Practice and Society, Institute of Education, University College London, London, United Kingdom; ^3^ Adolescent Girls and Young Women Department, Amani Girls Home, Mwanza, United Republic of Tanzania; ^4^ Tanganyika Christian Refugee Service, Shinyanga Unit, United Republic of Tanzania; ^5^ Department of Behavioral and Community Health, School of Public Health, University of Maryland, College Park, MD, United States; ^6^ Department of Behavioral and Community Health, Johns Hopkins School of Nursing, Baltimore, MD, United States

**Keywords:** transactional sex, age-disparate sex, gender-based violence (GBV), social norms intervention, mass media interventions, evaluation, mixed-methods, Tanzania

## Abstract

**Introduction:**

Age-disparate transactional sex (ADTS) is associated with HIV, unintended pregnancy, school dropout and violence, yet few interventions have successfully prevented it, and none have set ADTS prevention as their primary outcome. This exploratory evaluation aimed to assess indications of change after exposure to the Learning Initiative on Norms, Exploitation and Abuse (LINEA) intervention, a mass-media, gender-transformative social norms intervention aimed at preventing ADTS in Tanzania.

**Methods:**

In a condensed implementation 331 participants were instructed to listen to the LINEA radio drama over seven weeks, and 60 were randomly allocated to household discussion sessions about content. In-depth interviews (*n* = 81) from girls aged 12–16 years, and women and men caregivers were collected at baseline (September 2021), midline (November) and endline (December 2021). Surveys were conducted (*n* = 120) at baseline and endline using the Norms and Attitudes on ADTS Scale (NAATSS) and the Gender Roles and Male Provision Expectations (GRMPE) scale. Interviews were thematically analyzed using a framework approach. Age-stratified linear regression models adjusted for baseline scores were used to measure association between the intervention and endline scale scores.

**Results:**

Longitudinal data were available from 59 qualitative (73%) and 95 quantitative participants (79%). Qualitative evidence revealed the drama facilitated family conversations about adolescent challenges, allowing caregivers to advise daughters. Some girls gained confidence to refuse men's gifts, learning that accepting them could necessitate sexual reciprocation. Some caregivers felt increased responsibility for supporting girls in the community to avoid ADTS. Blame for ADTS shifted for some from girls to men, suggesting increased understanding of inequitable power dynamics and reductions in victim blaming. Marginal quantitative evidence revealed that highly exposed girls had improved gender equitable beliefs on the GRMPE (*β *= −6.26; 95% CI: −12.94, 0.42). Moderately exposed men had increased gender inequitable norms on the NAATSS subscale (*β *= 0.42 95% CI: 0.05, 0.79), but there was no effect in highly exposed men.

**Conclusions:**

Given the small sample results should be interpreted cautiously. Our initial findings indicate high engagement with the LINEA intervention shows promise in shifting knowledge, behaviors, and attitudes, beliefs and social norms driving ADTS in Shinyanga, Tanzania, supporting a robust impact evaluation.

## 1. Introduction

Age-disparate transactional sex, defined as a “non-commercial, non-marital sexual relationship motivated by the implicit assumption that sex will be exchanged for material support or other benefits” (1), is a well-established public health and human rights issue and a key contributor to the high HIV incidence among adolescent girls in sub-Saharan Africa (2). Adolescent girls and young women account for 32% of new HIV infections in Eastern and Southern Africa despite constituting only approximately 10% of the population (3) and are 2.5 times more likely to contract HIV than their male peers (4). Although HIV prevalence has been steadily decreasing in the region, reductions are significantly slower among adolescent girls and young women than other demographic groups (3, 5). Age-disparate transactional sex has also been associated with other negative sexual and reproductive health, developmental and social outcomes including early/forced marriage, sexual coercion, unplanned pregnancy, unsafe abortions, school dropout and violence (3, 4, 6–8).

The level of agency or exploitation in transactional sex relationships is mediated by complex, intersecting power inequalities based on age, gender, social status and access to resources (8–11). This study focuses on age-disparate transactional sex relationships between adolescent girls aged 12–16 years and men at least ten years older, which are intrinsically more exploitative because of these power inequalities. These inequalities also exacerbate negative health impacts, as once adolescent girls enter age-disparate transactional sex relationships their power to refuse sex or negotiate condom use reduces greatly (8).

This study builds on rich evidence on the role of social norms in driving transactional sex. Social norms are informal rules determining which behaviors are considered appropriate within a group (12–14), while attitudes and beliefs are situated at the individual level and can therefore be misaligned with norms (14, 15). Changing harmful norms is a non-linear process and requires long-term, consistent intervention (14). According to social norms theory, to effectively shift norms an intervention must change the beliefs and attitudes of enough individuals to reach a tipping point, demonstrating a “critical mass” in that group no longer approve of the norm (14, 16).

Findings from formative research by the Learning Initiative on Norms, Exploitation and Abuse (LINEA)^1^ in Brazil, Tanzania and Uganda show that for adolescent girls and young women the social norms driving their participation in transactional sex include community expectations that they should receive money, gifts or other benefits from sexual partners, that if they receive gifts they are expected to reciprocate sexually, and that they gain social status through ownership of material items (acquired through these relationships). For men, social norms driving participation in transactional sex include community expectations that men provide financially in sexual relationships, and that men should have a heightened sexuality and sexual prowess (8–10, 17–22).

Drawing on this formative research, LINEA developed a complex intervention aiming to shift the social norms driving age-disparate transactional sex in Tanzania using gender-transformative and synchronized approaches. The LINEA intervention is composed of two components: a radio drama and two curricula, one targeting adolescent girls and the other men. The intervention was co-developed between 2017 and 2020 by Amani Girls Home (AGH) an NGO, and behavior change communication specialists Media for Development International in Tanzania, and the London School of Hygiene & Tropical Medicine (LSHTM).

LINEA is the first intervention where preventing transactional sex is a primary outcome. It incorporates a gender and power lens and works with peer groups to critically reflect on short- and long-term implications of sexual transactions. A 2018 feasibility study conducted in Misungwi district, Mwanza region found the intervention acceptable, and contextually and culturally relevant (23). As a mass-media intervention, if found effective LINEA has the potential to be cost-effectively scaled up to impact entire populations (23). [For more information on LINEA intervention development and feasibility testing see (23)].

This exploratory study seeks to assess indications of change in adolescent girls and their caregivers following exposure to the LINEA intervention at the level of knowledge, behaviors, and attitudes, beliefs and social norms in Shinyanga, Tanzania. Thus, adolescent boys were excluded from this study. Findings on secondary outcomes and from a process evaluation, including evidence of unintended consequences, will be reported in forthcoming papers.

For the quantitative component we hypothesized the LINEA intervention would lead to participant's endorsing more gender-equitable beliefs and social norms related to age-disparate transactional sex. Based on social norms programming literature, we hypothesized this effect would be strongest amongst adolescent girls, women caregivers, and those living with a physical disability, and weakest amongst men caregivers and those without a physical disability (25–27).

This study contributes to the burgeoning global evidence on how entertaining mass-media or “edutainment” interventions can be used to prevent and reduce gender-based violence (e.g. 28, 29), in particular for hard-to-reach populations or where face-to-face implementation is challenging.

## 2. Methods

### 2.1. Study setting

This study was conducted in Kishapu district, Shinyanga region in northern Tanzania, located south of the Mwanza region and Lake Victoria. Evidence from Shinyanga points to entrenched gender inequitable norms and attitudes, including a culture of silence around sexual violence (30). This region has the highest rates of child marriage in Tanzania, with the Ministry reporting a 59% prevalence in 2017 (31). In a recent study, 35.9% of sexually active adolescent girls and young women reported experiencing any form of partner violence in the last six months (32).

### 2.2. Participants and sampling

Study participants were beneficiaries of the Tanganyika Christian Refugee Service (TCRS), an NGO working with the most vulnerable and marginalized populations in Kishapu. In 2020, TCRS distributed digital, solar powered radios to 331 households across 24 villages, all of which included a family member living with a disability, to share information through radio broadcasts about COVID-19, the impacts of climate change, gender-based violence and sexual and reproductive health.

Study participants were randomly sampled from TCRS beneficiary households and included adolescent girls aged 12–16 years, and women and men caregivers of adolescent girls within the same age range. Caregivers were defined as anyone who lived with and cared for an adolescent girl, including a parent, grandparent or sibling, among others. Caregivers were included to test the effects of the intervention on this group and determine whether their inclusion effected accessibility and impact of the radio drama on adolescent girls. Sampling occurred at the household level, and thus no participants were sampled from the same household; first for adolescent girls, then men caregivers followed by women caregivers. If there were multiple household members within the targeted demographic, participants were selected following decision trees based on study inclusion and exclusion criteria, and their willingness to partake (see Supplementary Material S1 for decision trees in English and Kiswahili).

Inclusion criteria were residing in Kishapu, being a recipient of a functional TCRS radio, being within the desired age range (12–16 years-old) or being a caregiver of an adolescent girl within the desired age range and being available to partake in study activities. If multiple household members met these criteria participants were selected using a coin toss. There were no exclusion criteria for caregivers based on age. Participants with a cognitive disability (as opposed to a physical disability) were excluded post-random selection (*n* = 57) because the intervention could not be reasonably expected to have the same impact on this population.

The final sample included 120 participants: 40 adolescent girls, 40 women caregivers and 40 men caregivers. Given the exploratory nature of the study, the sample size was not established to achieve a particular power or representativeness for the quantitative portion. Instead, the aim was to implement novel scales in a new context and among new populations, informing their use for a full evaluation. As this was a shortened implementation, we did not expect there to be large impacts detected in the quantitative data, but we nevertheless provide estimates of shifts in endline norms as they may be indicative of early change. The uncertainty around the point estimates of these shifts is reflected in the confidence intervals. For these reasons, the trends in the data we do observe must be interpreted cautiously, and in relation to the qualitative evidence, existing literature, and what we know about the intervention, with the goal of informing future implementation.

### 2.3. The intervention

#### 2.3.1. LINEA radio drama

The LINEA radio drama entitled “Msichana wa Kati” or “The Girl in the Middle” consists of 39 episodes (15–20 min each) designed to be played once per week over nine months on the radio. The drama was developed to help listeners critically reflect on the drivers of age-disparate transactional sex, and how the community can support adolescent girls and men to avoid it. “Msichana wa Kati” doesn't prescribe answers to sexual exploitation and abuse. Instead, the characters and storylines model positive arcs of change, highlighting the role of community members, including men, in supporting the central characters in changing harmful behaviors.

#### 2.3.2. Household discussion session

Trained TCRS facilitators delivered discussion sessions about radio drama content to assigned households once per week using discussion guides. These discussions covered key storylines and themes to promote critical reflection, including how caregivers can support their daughters to avoid age-disparate transactional sex, and how community members can support adolescent girls who have engaged in age-disparate transactional sex to mitigate potential harmful outcomes.

#### 2.3.3. Randomization

Using a random number generator, we randomized 20 participants of each demographic group to receive the radio drama only, and 20 to receive the radio drama and household discussions.

### 2.4. Data collection and procedures

All 331 TCRS beneficiary households were given the radio drama on USBs and instructed to listen over seven weeks to test a condensed version of the intervention. Giving participants USBs allowed them to listen at their own pace when convenient and allowed us to explore a novel delivery modality. TCRS community animators also delivered six discussion sessions to each household assigned.

Ten community animators were chosen to support this study from a larger pool of volunteers appointed by village assemblies based on their ability to read and write. In each of the 24 villages, two women and two men were selected to facilitate implementation. For this study, community animators underwent a two-week training led by AGH where they were introduced to the radio drama, discussion guides, study aims and methods. They were also tasked with ensuring only household members were present during discussion sessions to avoid “contamination” of participants not assigned.

Community animators facilitated AGH researcher's entry into villages. Most participants approached agreed to partake in the study because they wanted to learn how to support adolescent girls. Eleven participants declined, the most common reason being because they were more comfortable speaking the local language of Sukuma than Kiswahili, the language of the drama.

**Figure 1:**
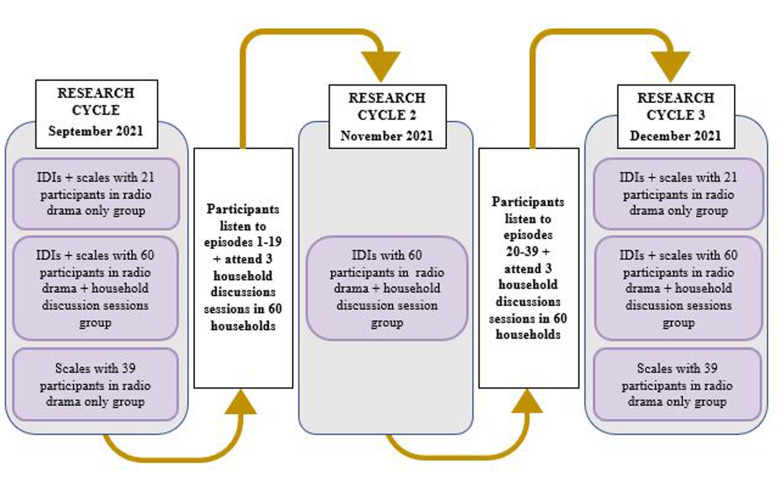
Diagram depicting data collection cycles of in-depth interviews (IDIs) and questions from the Norms and Attitudes on Age Disparate Transactional Sex scale (NAATSS) and the Gender Roles and Male Provision Expectations (GRMPE) scale.

120 participants (40 adolescent girls, women caregivers and men caregivers, respectively) completed baseline (September 2021) and endline (December 2021) questionnaires, and of these 81 were also randomly selected to partake in baseline and endline in-depth interviews (IDIs). Sixty midline IDIs (November) were also conducted with participants randomly allocated to partake in discussion sessions (see Figure 1 for a diagram depicting data collection cycles).

Data were collected by gender matched AGH researchers with technical support from LSHTM researchers, at a time and place convenient, private, and safe for participants. AGH have extensive experience working with adolescent girls on issues related to sexual health and gender-based violence and partook in LINEA formative research and feasibility testing. Prior to data collection AGH researchers also underwent a training in February 2021 and a refresher in May covering research ethics, informed consent and safeguarding, qualitative and quantitative research methods, data handling and processing, transcription and translation, researcher wellbeing and COVID-19 safety protocols.

#### 2.4.1. Qualitative tools

All interviews were conducted in Kiswahili using a semi-structured topic guide covering: (1) Experiences of disability and its impact on life, (2) Attitudes, beliefs and social norms linked to age-disparate transactional sex, (3) Experiences with age-disparate transactional sex, and (4) Experiences with and perceptions of the LINEA radio drama and discussion sessions. Topic guides were developed using experience and knowledge from formative research and feasibility testing, a review of the literature and based on study aims. AGH researchers contributed to the construction and ordering of questions, and piloted and refined them prior to data collection. IDIs were audio recorded and lasted between 45 and 90 min.

#### 2.4.2. Quantitative tools

##### 2.4.2.1. Outcome measures

Surveys were administered on mobile phones using Open Data Kit (ODK). Questions included demographics, school enrolment (for adolescent girls), the Washington Group Short Set on Functioning to assess physical disability (33), and questions from two scales, which made up our five outcome measures listed below.

**Scale 1**. The Norms and Attitudes on Age Disparate Transactional Sex Scale (NAATSS), which measures our primary outcome. We considered:

(1) The total NAATSS score
(2) The NAATSS Attribution to Girls' behavior subscale
(3) The NAATSS Men's Motivations subscale
(4) The NAATSS Girls' Readiness to Have sex subscale

The NAATSS was developed from LINEA formative research in Brazil to measure social norms that justify and/or foster age-disparate transactional sex (34). In this study, we adapted the NAATSS questions from the Attribution to Girls' Behavior subscale that assess adolescent girls' reasons for engaging in age-disparate transactional sex and their behaviors that are perceived to be linked to their engagement. The Men's Motivations subscale that assesses the role men's pleasure and power play in their engagement in age-disparate transactional sex. As well as the Girls' Readiness to Have Sex subscale that assesses whether an adolescent girls' physical development or age are seen as signifying their sexual readiness, and whether age-disparate transactional sex can benefit adolescent girls and men. Cronbach's alphas in this dataset were 0.85 for the NAATSS and 0.77, 0.83 and 0.70 for the subscales, respectively (see Supplemental Material S2 for the NAATSS questions in English and Kiswahili).

All outcomes were treated as continuous variables, and we calculated summary scores for all scales and subscales. For the total NAATSS possible scores ranged from 13 (most gender equitable) to 52 (least gender equitable). For the Attributions to Girls' Behavior and Men's Motivations subscales possible scores ranged from 5 (most gender equitable) to 20 (least gender equitable), while for the Girl's Readiness to have Sex subscale possible scores ranged from 3 (most gender equitable) to 12 (least gender equitable).

**Scale 2.** The Gender Roles and Male Provision Expectations (GRMPE) scale, which measures beliefs, a proxy for the primary outcome. We considered:

(5) The total GRMPE score

For this exploratory study we conceptualize beliefs measured by the GRMPE as a proxy for norms as changes in personal beliefs indicate the potential beginning of social norms change, and given the short implementation and limited sample size, changes at the individual level were more likely.

Stoebenau and colleagues developed the GRMPE in Central Uganda to capture beliefs linked to male provider role expectations. The scale's objective is to assess whether adherence to beliefs about men's and women's gender roles in the context of male provider role expectations may contribute to adolescent girl and young women's increased risk of HIV, particularly within the context of transactional sex relationships (35, 36). We adapted two GRMPE subscales for this study. The Male Provision and Sexual Decision-making subscale which includes six items that assess whether participants agree that when a man is providing material support to a partner, he should have more control over sexual decision-making. An example of an item is: “*If a man is providing a lot of financial support (to his partner) then he should decide whether or not they use a condom during sex.*” The Male Provision and Sexual Agency subscale includes six items that assess whether participants agree with the belief that women should use their sexuality to gain material support from men. One item states, “*As a woman, it's important to know how to use physical beauty to get whatever you want from a man.*” The Cronbach's alpha in this dataset was 0.82.

For the GRMPE possible total scores ranged from 12 (most gender equitable) to 48 (least gender equitable). For all outcome measures decreased scores between baseline and endline were the desirable direction of change.

This study is the first to use these novel scales in Tanzania, and with men in the case of the GRMPE, making an important contribution to their adaptation and validation. AGH researchers, all of whom are fluent in Kiswahili and English, translated both scales into Kiswahili and adapted them for use with adolescent girls. They piloted and further refined them prior to data collection.

##### 2.4.2.2. Exposures

Participants were asked at endline: “*How many of the 39-episodes of the “Msichana wa Kati’ radio drama did you listen to*?” Answer options were: 1. None, 2. Some, 3. Half, 4. Almost All and 5. All. participants were also asked how many discussion sessions they attended from 0 to 6.

### 2.5. Data analysis

#### 2.5.1. Thematic analysis

IDIs were translated and transcribed verbatim into English. We developed a thematic coding framework *a priori* based on topic guides and previous research on age-disparate transactional sex in sub-Saharan Africa (1, 8). We coded a few transcripts from adolescent girls, women caregivers and men caregivers using NVivo 12, and collaboratively adapted and refined the framework based on coder discrepancies. We then coded the remaining transcripts, double coding 20% of the richest transcripts to ensure consistency and reliability. Once completed, we returned to parent codes and re-read the data, developing child codes as they emerged. We further analyzed findings to assess strength of evidence and compare between adolescent girls, women caregivers and men caregivers.

We used a framework approach to analyze participant data longitudinally (37). We recorded data in a matrix with columns representing key themes and rows representing each participant's responses at each time point. In the subsequent row we recorded observed changes in participants' responses over time. We discussed preliminary findings in an interpretation meeting with the PI, AGH and TCRS teams, contextualizing them and adding nuance. In reporting our results, we draw on data from close to half of participants, highlighting a breadth of experiences and perceptions. We report on key thematic findings, and counter evidence where available.

#### 2.5.2. Statistical analysis

We conducted an exploratory analysis using linear regression models to understand the strength of association between the intervention and endline beliefs and social norms. All models were adjusted for baseline scale scores (and thus time between baseline and endline) and age.

We first conducted an intention-to-treat analysis to compare changes in outcomes over time between participants assigned to the radio drama only vs. radio drama and discussion sessions. Given the exploratory nature of this study, and our interest in uncovering the effects of each component of the intervention, we then conducted a per-protocol analysis based on participant's self-reported exposure to the drama, and household discussion session implementation data (to account for inconsistencies in the self-reported data). We created binary exposure variables and considered three categorizations of exposure:

(a) Radio drama: Exposed if individuals listened to half or more of the drama; unexposed otherwise.
(b) Household discussion sessions: Exposed if individuals attended at least one discussion session; unexposed otherwise.
(c) Combination: “High” if individuals were exposed to both the drama and discussion sessions as above; “moderate” if individuals were exposed to either the drama or discussion sessions as above; unexposed otherwise.

Two sets of models were planned: The first were fit separately in adolescent girls, women caregivers and men caregivers to account for *a priori* hypothesized effect modification by demographic. The second were fit separately in individuals who reported a disability and those who didn't to account for potential differential exposure access.

### 2.6. Ethical considerations

Ethical considerations were essential for this study given the sensitivity of the topic and the inclusion of “vulnerable” populations, such as young adolescent girls and people living with disabilities. All adolescent participants provided written informed assent, and their caregiver and adult participants provided written informed consent. LSHTM and AGH safeguarding policies and procedures for conducting research with children and vulnerable populations were followed, and AGH managed all reported safeguarding cases, which were referred to local services and safe housing as appropriate.

Strict adherence to General Data Protection Regulation (GDPR) guidelines were followed to protect confidentiality and anonymity, such as using unique identifiers and storing data in encrypted, password protected devices and platforms. This study was approved by the National Health Research Ethics Committee (NaTHREC) of the National Institute for Medical Research (NIMR) in Tanzania (Ref: NIMR/HQ/R.8a/Vol.IX/3698) and the Ethics Committee of LSHTM (ref: 22863-1). A Data Transfer Agreement for this study was approved by NIMR.

## 3. Results

In this section we describe study participants characteristics, and then present our qualitative findings on indications of change following intervention exposure, comparing results between adolescent girls, women caregivers and men caregivers at baseline, midline and endline. We conclude with our quantitative findings, interpreting them within the context of our qualitative results.

### 3.1. Study participant characteristics

Longitudinal qualitative data were available for 59 participants (73%): 23 adolescent girls, 18 women caregivers and 18 men caregivers. Midline data were available for 45 participants (76% of those allocated), for a total of 163 transcripts. The mean age was 14 years for adolescent girls, 46 years for women, and 56 years for men (Table 1). Participants who partook in IDIs comprised a subset of the quantitative sample.

**Table 1:** Characteristics of qualitative study participants.

	All *N* (%)	Adolescent girls *n* (%)	Women caregivers *n* (%)	Men caregivers *n* (%)
Total number	59	23 (39)	18 (31)	18 (31)
Household discussion sessions (midline data)	45 (76)	16 (70)	16 (89)	13 (72)
No household discussion sessions (no midline data)	14 (24)	7 (30)	2 (11)	5 (28)
Mean age at baseline (range, in years)	37 (12–76)	14 (12–16)	46 (31–68)	56 (25–76)

Follow-up quantitative data were available for 95 participants (79%): 34 adolescent girls, 31 women and 30 men. The primary reasons for participant drop out was being away from home for income generating activities, and busy after repeated visits. One adolescent girl was married and moved away, and one man passed away. The majority of adolescent girls were 15 years (*n* = 19). Adult participants ranged from 25 to 85 years. The mean age for men was 52 years, and for women 48 years. Seven participants reported living with a disability (one adolescent girl and six women). All adolescent participants were in school at baseline, and two reported leaving by endline (Table 2).

**Table 2:** Characteristics of quantitative study participants.

	All *N* (%)	Adolescent girls *n* (%)	Women caregivers *n* (%)	Men caregivers *n* (%)
Total number	95	34 (36)	31 (33)	30 (32)
Age at baseline, in years: Mean (SD); range	37.1 (20.5); 12–85	14.2 (1.1); 12–16	48.0 (14.5); 27–76	51.7 (13.7); 25–85
Disability:				
Yes	7 (7)	1 (14)	6 (86)	0 (0)
None	88 (93)	33 (38)	25 (28)	30 (34)

### 3.2. Qualitative results

Here we present thematic findings that emerged from IDIs with adolescent girls, women caregivers and men caregivers. We begin by describing participant's reported intervention exposure, and then present findings on indications of change following exposure related to age-disparate transactional sex.

#### 3.2.1. Exposure to the LINEA intervention

The majority of participants reported listening to the entire radio drama by endline, with only six reporting listening to less than half, and one woman reporting listening to none because she was caring for an ill family member. Household discussion session attendance was more evenly split, with approximately half of participants reporting having attended one or more.

#### 3.2.2. Indications of change following the LINEA intervention

Over half of participants reported the intervention had a desirable impact related to age-disparate transactional sex. We also found important changes in how adolescent girls, and women and men caregivers responded to questions between baseline and midline and/or endline, although participants didn't always explicitly attribute this change to the intervention. These self-reported and observed indications of change are further described in the following sections on knowledge, behaviors, and attitudes, beliefs and social norms.

##### 3.2.2.1. Knowledge

###### 3.2.2.1.1. What age-disparate transactional sex is and its consequences

A few adolescent girls reported not knowing what transactional sex was at baseline but demonstrated a greater understanding of these relationships and how they start at endline. For example, one adolescent girl reported at baseline she didn't know how age-disparate transactional sex relationships began, but believed they ended in marriage. When asked the same questions at endline she said:

*The man starts by offering her gifts… He will ask for his things/money back and the girl will not have [them], hence he will suggest to have sex with [him] to pay for it, and she will accept. When she gets pregnant, the man will leave her.* (Adolescent girl aged 14-years, endline, IDI 13)

At endline this adolescent girl demonstrated more accurate knowledge of the consequences of age-disparate transactional sex, which often does not end in marriage. Like this adolescent girl, several others also reported an increased understanding that accepting gifts from men could lead to the expectation they must reciprocate with sex, which could result in pregnancy. When asked if an adolescent girl who accepted gifts would have to give something in return, one participant responded at baseline she could show her gratitude by simply “*saying thank you*”, while at endline she responded: “*the person who gave her things, he will ask for them back, if she doesn’t have them, she will enter into transactional sex.*” (Adolescent girl aged 13-years, endline, IDI 11).

Women and men caregivers also demonstrated a more holistic knowledge of risks associated with age-disparate transactional sex between baseline and endline. For example, a man who initially only suggested sexually transmitted infections as a risk for adolescent girls, at endline said: “*Apart from pregnancy and diseases… Her mind is shifted to sexual issues, instead of thinking about studies and other important things in life… she will continue with that behavior because she started sexual issues early*.” (Man aged 59-years, caregiver, endline, IDI 51). This participant identified long-term consequences of engaging in age-disparate transactional and recognized more mental and sexual health implications for adolescent girls at endline compared to baseline.

###### 3.2.2.1.2. Challenges faced by adolescent girls

There was strong evidence that many caregivers became more aware of the challenge's adolescent girls faced in the community through listening to the drama:

*It [the radio drama] was a lesson… for us parents to learn what our girls pass through when they are at school or on the way…It [the radio drama] changed my family. I was in the dark, I didn't know they were being abused but now I am aware of it.* (Woman aged 38-years, caregiver, endline, IDI 95)

Adolescent girls reported that because their caregivers were more aware of their challenges, they could now support them in overcoming them. For example, an adolescent girl said: “*if all of us have understanding parents, then […] we will not face any challenges*.” (Adolescent girl aged 15-years, endline, IDI 7).

##### 3.2.2.2. Behaviors

###### 3.2.2.2.1. Family conversations

Closely linked to the indications of change seen in participant's knowledge, there was also strong evidence from adolescent girls, and women and men that listening to the radio drama together sparked family conversations about challenge's adolescent girls face, giving caregivers the opportunity to advise their daughters:

*We talked about the boda-boda [motorcycle taxi] drivers giving girls lifts when they are going to school… I always switched on the radio for them [her daughters] to listen to the drama… and while listening together I got the time to tell them to stop that behavior [accepting lifts].* (Woman aged 32-years, caregiver, midline, IDI 87)

*Many times we were listening to the radio drama, and… I told the people in our group [family] to stop playing the radio drama and I asked them: “between the characters in the radio drama, which character has given good advice?”* (Man aged 57-years, caregiver, endline, IDI 76)

Thus, the drama was seen as a positive influence by participants, offering them the opportunity for reflection and discussion. There was evidence it also provided context and language to facilitate these conversations as adolescent girls and caregivers could refer to situations and characters in the drama to discuss what was happening in the community. For example, a central storyline is 13-year-old Amali being offered “free” rides to school by Tuma, a motorcycle taxi driver in his 20s:

*When they [his granddaughters] came back from school, they said “Grandfather, the things happening between Tuma and Amali in the radio drama, we are experiencing the same on our way to school” … So, I told them, “When you meet with people, and they start saying things like what we are hearing in this radio drama, you have to say no!”* (Man aged 76-years, caregiver, endline, IDI 48)

This demonstrates how the drama gave caregivers and daughters a reference point, and impetus, to discuss adolescent challenge's, in this case being offered “free” rides. It gave adolescent girls the opportunity to share their experiences with caregivers, opening bigger conversations. Adolescent girls reported feeling relieved talking to their caregivers about their challenges. For example, one adolescent girl said:

*They [parents] didn't know [the challenges faced by girls]… so, it was good for them to know… us as a family we need to live like the radio drama and to copy it… [parents should] sit with their girls and talk to them about men and transactional sex on the road.* (Adolescent girl aged 13-years, endline, IDI 34)

This reflects how the drama not only warned listeners of the risks of age-disparate transactional sex, but also encouraged them to adopt modelled behaviors. Other adolescent girls also reported when discussing the drama with their caregivers they were instructed to tell them if men harassed them in the future, or to ask for money if they needed it.

###### 3.2.2.2.2. Saying no to men

Some adolescent girls reported that through the radio drama they gained the confidence to decline men who offered them gifts or asked for sex. Rejecting men was particularly challenging for adolescent girls in this context where elders, and particularly men, are revered, and children are taught to respect their authority. Thus, declining or disobeying a man could appear disrespectful. Conversely, accepting a man's gifts or request for sex could also reflect negatively on adolescent girls, as highlighted by one woman: “*The girls do not know how to refuse men. Even if they don’t want to be with the man, they find themselves in that situation. So, the people think they have bad behavior.*” (Woman aged 38-years, caregiver, midline, IDI 95).

Adolescent girls reported learning to confidently turn down men through the character of Mama Prita, an older role model for the adolescent girl characters who advised them about healthy relationships: “*Mama Prita… she tells us girls to say no with courage to the men who follow us.*” (Adolescent girl aged 14-years, endline, IDI 13). Hence, Mama Prita reportedly advised adolescent girl listeners in addition to the characters.

Some caregivers, especially women, also reported learning from Mama Prita how to teach their daughters to reject men, reiterating this lesson to them. A few caregivers highlighted, however, that it could be challenging for adolescent girls to learn and required repeated conversations, starting at a young age:

*The hardest part is to teach her… [she is] just a girl… The girl is young so you [must] go back to her from time to time… [And tell her] “stop [accepting gifts from older men] because you are still young” … the situation is hard… teaching a little girl.* (Man aged 53-years, caregiver, endline, IDI 55)

This highlights the important of repeated conversations between caregivers and young adolescent girls about the risks of age-disparate transactional sex, also suggesting adolescent girls could benefit from repeated listening to the drama, and caregivers could help maintain the messages after the intervention concludes.

###### 3.2.2.2.3. Age-disparate transactional sex

No participants reported having engaged in age-disparate transactional sex themselves, but most agreed it happened occasionally or often in their communities, and many shared stories of friends or neighbors who had. A few adolescent girls reported being offered money, gifts, or rides to school from older men, and two accepted, but none reported reciprocating with sex. Only one man reported having given an adolescent girl a gift, but this had been when he was young himself (Older man, age unknown, caregiver, baseline and endline, IDI 50).

##### 3.2.2.3. Attitudes, beliefs and social norms

###### 3.2.2.3.1. Responsibility for initiating age-disparate transactional sex relationships

Most caregivers believed men were ultimately responsible for initiating age-disparate transactional sex relationships because they had the power to manipulate adolescent girls. Among the few caregivers who saw adolescent girls as sometimes responsible for initiating the relationships, we saw an increased understanding of the responsibility of men between baseline and midline and/or endline. For example, a man who at baseline said age-disparate transactional sex starts because “*she meets a guy willing to offer her a ride, but she must also give something in return… The family is poor.*” (Man aged 42-years, caregiver, baseline, IDI 49), at endline said:

*They [older men] give her some gift, money, maybe transport… At the end of the day, it becomes a normal situation for that child, so it reaches a time, a man starts to introduce his issue [sex]… Girls, they are being approached by men, they are being put in a trap, at the end of the day they are captured.* (Man aged 42-years, caregiver, endline, IDI 49)

While this participant recognized power inequalities based on access to money, by endline he describes age-disparate transactional sex as a “*trap*”, demonstrating an understanding of additional power inequalities rooted in age and development.

Among adolescent girls we saw the strongest evidence of a shift from blaming adolescent girls for initiating these relationships at baseline, to blaming men at endline. For example, at baseline an adolescent girl said:

*Let's say a girl like me starts asking for money from that man every day […] At the end the man will ask himself why she is asking me for the money, let me try and seduce her. Then when he seduces her, she accepts.* (Adolescent girl aged 15-years, baseline, IDI 123).

In contrast, when asked the same question at endline she replied,

*When you meet the man on the way and he gives you money, sweets or lifts, they start like that, and later on you will regret it.* (Adolescent girl aged 15-years, endline, IDI 123).

This demonstrates an increased understanding of the inequitable power dynamics between adolescent girls and men, which may also indicate a reduction in peer stigmatization and victim blaming attitudes towards adolescent girls who have been in age-disparate transactional sex relationships.

###### 3.2.2.3.2. Responsibility for supporting adolescent girls

There was evidence from adolescent girls, and women and men that after the intervention caregivers took on a greater responsibility for supporting their daughters to avoid risky behaviors. For example, a man said:

*The radio drama aims [to teach] … that we should not abandon girls… When they face challenges from their daily activities, we should be at the frontline educating them.* (Man aged 46-years, caregiver, endline, IDI 47)

Many caregivers, especially women, reported learning about the importance of parent-daughter connectedness when providing this support. As one man put it: “*we have to first build friendships with children*” (Man aged 25, caregiver, endline, IDI 43). When asked what she learned from the drama another participant replied:

*[I learned through the radio drama that] our girls, we need to be close with them, and educate them on transactional sex. This is the problem, she thinks she is given [gifts from men] for free, but it's expensive.* (Woman aged 46-years, caregiver, endline, IDI 83)

The evidence suggests increased caregiver support extended beyond prevention of age-disparate transactional sex, to caring for adolescent girls experiencing negative consequences from it. For example, one man reported learning that if his adolescent daughter were to become pregnant, he should take legal action against the man who impregnated her (Man aged 53-years, caregiver, endline, IDI 55), while a few mothers reported learning they should continue to look after their daughters:

*If they [the parent characters] didn't chase their daughter away [when she became pregnant] […] then they would be at peace. We need to be close to our children… Take care of her until she has a baby. We don't need to desert them.* (Woman aged 33-years, caregiver, endline, IDI 97)

In line with the aims of the intervention, evidence also emerged from adolescent girls, and women and men caregivers that the drama reinforced that adolescent girls should support each other to avoid age-disparate transactional sex. For example, an adolescent girl said:

*Because others got pregnant while they were at school… [my mother] told me I am supposed to advise my fellow students not to accept gifts [because] they can [experience negative] consequences.* (Adolescent girl aged 13-years, endline, IDI 20)

###### 3.2.2.3.3. Social norms:

We found limited evidence of social norms change, likely due to the short implementation period and limited number of participants. For example, at baseline we found adolescent girls and men who engage in age-disparate transactional sex were perceived by the community as “*prostitutes*” and having “*bad behavior*”, and no meaningful change was detected at endline.

We did, however, find evidence of change in a few caregivers' perceived sense of responsibility for protecting adolescent girls in the community from age-disparate transactional sex. For example, when asked how she changed after listening to the radio drama a woman replied: “*even the neighbor's daughter is my daughter. I should help when needed*.” (Woman aged 46-years, caregiver, endline, IDI 83). Similarly, when a man was asked if the drama changed his dreams for his community he responded:

*[The radio drama] has made me change… I should be seeing the actions of those young girls [in the community] and criticizing them as they are not doing right… I [should] put them right. That radio has […] boosted my energy [to help adolescent girls].* (Man aged 63, caregiver, endline, IDI 75)

There were a few caregivers who expressed apathy about being able to prevent age-disparate transactional sex at baseline, but whose expressed attitudes changed at endline. For example, one woman who said you can do “*nothing at all*” at baseline “*because you already advise them [adolescent girls] well but when they go out, they are going to do their things, what can you tell them again?*”, at endline advised community members: “*protect each other, if a man see's other girls he must treat her like his daughter and he should protect her*.” (Woman aged 58-years, caregiver, baseline and endline, IDI 91).

These changes highlight a shift away from blaming adolescent girls for participating in age-disparate transactional sex, to laying the burden of responsibility on men and the community. Moreover, they suggest the potential beginning of social norms change, as caregivers shift to caring for all adolescent girls in the community as their own daughters, making it a communal responsibility to protect adolescent girls from age-disparate transactional sex.

### 3.3. Quantitative results

Our quantitative results supported some of our qualitative findings on indications of change in beliefs (measured by the GRMPE) and added some additional insights on social norms change (measured by the NAATSS and its subscales) related to age-disparate transactional sex. Given the small sample size, however, especially within each demographic group, these results must be interpreted with caution.

At baseline men caregivers had the most gender inequitable scores and adolescent girls had the most gender equitable scores across all scales and subscales (Table 3). Table 4 presents outcomes from our intention-to-treat analysis, where we found no intervention effect in adolescent girls or men, but weak evidence of increased gender inequitable norms in women on the NAATSS Girl's Readiness to Have Sex subscale (*β* = 0.29; 95% CI: −0.02, 0.60). This finding suggests women caregivers who were assigned to attend household discussion sessions were more likely to agree with norms related to girls' readiness to have sex being based on her physical development or age at endline compared to baseline.

**Table 3:** Baseline and endline scores on the Norms and Attitudes of Age Disparate Transactional Sex scale (NAATSS) and the Gender Roles and Male Provision Expectations (GRMPE) scale among adolescent girls, women caregivers and men caregivers.

	All *N* = 95	Adolescent girls *n* = 34	Women caregivers *n* = 31	Men caregivers *n* = 30
NAATSS baseline: Mean (SD)				
All subscale total scores; average scores	35.5 (0.6); 2.7 (0.4)	33.6 (1.2); 2.6 (0.1)	35.8 (0.9); 2.8 (0.1)	37.4 (0.7); 2.9 (0.1)
Attribution to Girls’ Behavior	2.7 (0.1)	2.6 (0.1)	2.7 (0.1)	2.9 (0.1)
Men's Motivation	2.9 (0.1)	2.7 (0.1)	3.0 (0.1)	3.0 (0.1)
Girls’ Readiness to Have Sex	2.6 (0.1)	2.5 (0.1)	2.5 (0.1)	2.6 (0.1)
GRMPE baseline: Mean (SD)				
All subscales total scores; average scores	29.7 (0.6); 2.5 (0.0)	26.3 (1.1); 2.2 (0.1)	30.9 (0.8); 2.6 (0.1)	32.2 (0.8); 2.7 (0.1)
NAATSS endline: Mean (SD)				
All subscales total scores; average scores	37.1 (0.7); 2.9 (0.1)	36.8 (1.3); 2.8 (0.1)	38.3 (1.2); 2.9 (0.1)	36.1 (0.8); 2.8 (0.1)
Attribution to Girls’ Behavior	2.8 (0.1)	2.9 (0.1)	2.9 (0.1)	2.7 (0.1)
Men's Motivation	3.0 (0.1)	3.0 (0.1)	3.1 (0.1)	3.0 (0.1)
Girls’ Readiness to Have Sex	2.6 (0.1)	2.5 (0.1)	2.7 (0.1)	2.5 (0.1)
GRMPE endline: Mean (SD)				
All subscales total scores; average scores	30.7 (0.7); 2.6 (0.1)	28 (1.6); 2.3 (0.1)	32.6 (1.1); 2.7 (0.1)	31.9 (0.8); 2.7 (0.1)

**Table 4:** Intention-to-treat effect of the LINEA intervention on the Norms and Attitudes of Age Disparate Transactional Sex scale (NAATSS) and the Gender Roles and Male Provision Expectations (GRMPE) scale among adolescent girls, women caregivers and men caregivers at endline.

		NAATSS endline				GRMPE endline
	Household discussion sessions	Total	Attribution to Girls’ Behavior	Men's Motivation	Girls’ Readiness to Have Sex	Total
Adolescent girls	Not assigned	REF	REF	REF	REF	REF
	Assigned	−1.78 (−7.70, 4.15)	−0.07 (−0.54, 0.40)	−0.24 (−0.80, 0.31)	−0.07 (−0.63, 0.49)	−0.94 (−9.49, 6.60)
Women caregivers	Not assigned	REF	REF	REF	REF	REF
	Assigned	3.24 (−1.63, 8.10)	0.36 (−0.10, 0.82)	0.18 (−0.30, 0.67)	**0.29 (-0.02, 0.60)**	3.33 (−1.20, 7.86)
Men caregivers	Not assigned	REF	REF	REF	REF	REF
	Assigned	0.08 (−3.62, 3.78)	0.04 (−0.38, 0.46)	0.05 (−0.27, 0.38)	−0.16 (−0.58, 0.25)	−0.25 (−3.55, 3.05)

Bold values indicate *p* < 0.10.

Thirty-four participants (36%) self-reported listening to at least half of the radio drama and 43 (72% of those allocated) attended at least one discussion session. In total, 29 participants were categorized as having “high” (31%), 35 as “moderate” (37%) and 31 as “no or low” (33%) combined intervention exposure. Adolescent girls, and women and men caregivers were relatively evenly distributed across these groups (Table 5).

**Table 5:** Quantitative study participant's self-reported exposure to the LINEA radio drama, and implementation data on exposure to household discussion sessions.

	All *N* (%)	Adolescent girls *n* (%)	Women caregivers *n* (%)	Men caregivers *n* (%)
Radio drama exposure				
Less than half of episodes	61 (64)	24 (71)	18 (58)	19 (63)
Half or more episodes	34 (36)	10 (29)	13 (42)	11 (37)
Discussion session exposure				
None (0 sessions)	52 (55)	20 (59)	13 (42)	19 (63)
Some (1-6 sessions)	43 (45)	14 (41)	18 (58)	11 (37)
Combined intervention exposure				
None or low	31 (33)	13 (38)	10 (32)	8 (27)
Moderate	35 (37)	11 (32)	11 (36)	13 (43)
High	29 (31)	10 (29)	10 (32)	9 (30)

Results from our per-protocol analysis (Table 6) revealed weak evidence of impact of the radio drama in adolescent girls on the GRMPE (*β *= −6.26; 95% CI: −12.94, 0.42). In line with the qualitative evidence, this finding suggests for this group listening to half or more of the radio drama was associated with more gender equitable beliefs that were contrary to the expectation that men should provide financially, and women should reciprocate with sex.

**Table 6:** Per-protocol effect of the LINEA intervention on the Norms and Attitudes of Age Disparate Transactional Sex scale (NAATSS) and the Gender Roles and Male Provision Expectations (GRMPE) scale among adolescent girls, women caregivers and men caregivers at endline.

		NAATSS endline				GRMPE endline
	Total	Attribution to Girls’ Behavior	Men's Motivation	Girls’ Readiness to Have Sex	Total
Radio drama exposure						
Adolescent girls	Less than half	REF	REF	REF	REF	REF
	Half or more	−1.71 (−7.25, 3.83)	0.10 (−0.35, 0.55)	−0.26 (−0.78, 0.25)	−0.31 (−0.86, 0.24)	**−6.26 (−12.94, 0.42**)
Women caregivers	Less than half	REF	REF	REF	REF	REF
	Half or more	−0.32 (−5.85, 5.21)	0.11 (−0.41, 0.62)	−0.11 (−0.60, 0.39)	0.11 (−0.23, 0.45)	1.41 (−2.96, 5.79)
Men caregivers	Less than half	REF	REF	REF	REF	REF
	Half or more	2.81 (−0.92, 6.53)	**0.35** **(−0.06, 0.77)**	0.22 (−0.11, 0.56)	−0.11 (−0.53, 0.32)	0.66 (−2.79, 4.11)
Discussion session exposure						
Adolescent girls	None	REF	REF	REF	REF	REF
	One or more	−2.09 (−7.90, 3.71)	−0.13 (−0.59, 0.33)	−0.22 (−0.77, 0.34)	−0.09 (−0.65, 0.47)	−1.89 (−9.53, 5.75)
Women caregivers	None	REF	REF	REF	REF	REF
	One or more	2.74 (−2.41, 7.89)	0.24 (−0.24, 0.72)	0.24 (−0.25, 0.72)	0.26 (−0.08, 0.60)	1.04 (−3.31, 5.40)
Men caregivers	None	REF	REF	REF	REF	REF
	One or more	−1.56 (−5.62, 2.49)	−0.24 (−0.69, 0.20)	−0.03 (−0.38, 0.33)	−0.06 (−0.49, 0.37)	−0.84 (−3.98, 2.31)
Combined exposure						
Adolescent girls	No or low	REF	REF	REF	REF	REF
	Moderate	0.32 (−7.03, 7.66)	0.14 (−0.45, 0.73)	−0.10 (−0.76, 0.56)	0.04 (−0.67, 0.75)	−4.65 (−14.21, 4.92)
	High	−2.67 (−9.79, 4.44)	−0.01 (−0.59, 0.57)	−0.35 (−1.01, 0.30)	−0.30 (−1.00, 0.40)	−7.36 (−16.87, 2.15)
Women caregivers	No or low	REF	REF	REF	REF	REF
	Moderate	1.27 (−5.35, 7.90)	0.35 (−0.28, 0.98)	−0.16 (−0.79, 0.48)	0.31 (−0.13, 0.75)	3.14 (−2.24, 8.52)
	High	2.15 (−4.90, 9.20)	0.29 (−0.34, 0.91)	0.08 (−0.54, 0.70)	0.34 (−0.11, 0.78)	2.05 (−3.47, 7.57)
Men caregivers	No or low	REF	REF	REF	REF	REF
	Moderate	1.45 (−3.07, 5.97)	0.04 (−0.48, 0.56)	**0.42** **(0.05, 0.79)**	−0.38 (−0.86, 0.10)	1.67 (−1.97, 5.31)
	High	1.21 (−3.97, 6.40)	0.10 (−0.47, 0.68)	0.19 (−0.23, 0.60)	−0.14 (−0.66, 0.39)	−0.29 (−4.45, 3.86)

Bold values indicate *p* < 0.10.

A result novel to the quantitative evidence was that amongst men caregivers moderate combined intervention exposure was associated with increased gender inequitable norms scores on the NAATSS Men's Motivations subscale (*β* = 0.42; 95% CI: 0.05, 0.79), but this effect was not present among highly exposed men (*β* = 0.19; 95% CI: −0.23, 0.61). This suggests men who listened to less than half of the radio drama or didn't attend any discussion sessions were more likely to ascribe to norms about men gaining power and pleasure from engaging in age-disparate transactional sex at endline compared to baseline.

We didn't run the planned second set of models stratified by disability due to the small sample of participants reporting a disability (*n* = 7) (Table 1).

## 4. Discussion

The LINEA intervention shows promise in shifting knowledge, behaviors, attitudes and beliefs driving age-disparate transactional sex in Shinyanga, Tanzania. Indications of change were mostly evident in the longitudinal qualitative data, with changes in adolescent girls' beliefs additionally supported by preliminary quantitative evidence. We also found qualitative indications of future social norms change.

In this section we begin by summarizing the key qualitative and quantitative findings, followed by the strengths and limitations of this study, and concluding with its implications.

### 4.1. Key findings

We found robust qualitative evidence of the intervention shifting adolescent girls', and women and men caregivers' knowledge, behaviors, attitudes and beliefs related to age-disparate transactional sex. For instance, adolescent girls who had listened to the radio drama reported increased confidence to refuse gifts from men, and realized that accepting these gifts signaled a willingness to reciprocate with sex. This was collaborated by the quantitative data, which showed that adolescent girls' who listened to half or more of the drama were more likely to disagree that men who provide financially for their partner should be able to control sexual decision-making compared to their views before being exposed to the intervention. These findings are in line with messages from the drama, in which the adolescent girl characters learn to refuse sex with men who offer them gifts. It is notable we only saw this desirable quantitative change in adolescent girls even though they listened to less of the drama than women and men (Table 5), suggesting the drama strongly resonated with these adolescent girls. These results are also consistent with existing literature suggesting that norm change in adults may be more challenging than with adolescents due to rigid attitudes and beliefs (25).

Moreover, longitudinal qualitative evidence showed that adolescent girls learned about the risks associated with age-disparate transactional sex. At endline some caregivers gained a more nuanced understanding of the long-term consequences of adolescent engagement in these relationships compared to baseline. Caregivers also reported increased knowledge of the challenges their daughters faced, promoting caregiver support for adolescent girls and encouraging parent-daughter connectedness, which has been found to help prevent age-disparate transactional sex (38, 39).

Although we did not observe meaningful changes in social norms in the qualitative data—likely due to the short implementation period and small sample—we found indications of potential future norms change. For instance, we found evidence of a few caregivers increased perceived responsibility for supporting all adolescent girls in their community in avoiding age-disparate transactional sex as they would their own daughters. Some participants also demonstrated a shift from blaming adolescent girls for starting age-disparate transactional sex relationships at baseline to blaming men at endline. This suggests an increased understanding of the power inequalities between adolescent girls and men rooted in age and development, and a shift away from stigmatization and victim blaming attitudes, potentially representing the beginning of gender-transformative change.

Quantitatively, we found weak evidence of increased gender inequitable norms amongst women assigned to discussion sessions, who were more likely to agree with social norms related to girls' readiness to have sex being based on her physical development or age at endline compared to baseline (Table 4). Similarly, men caregivers with moderate intervention exposure were more likely to ascribe to harmful norms about men gaining power and pleasure from engaging in age-disparate transactional sex compared to baseline, but this effect was not present in men with high intervention exposure. This could be explained by the men characters in the drama experiencing an “arc of change”: at the beginning they held gender inequitable attitudes and behaviors, but they changed over the course of the story. Therefore, men with moderate intervention exposure (attending no household discussions, or only listening to some drama episodes) were more likely to have been exposed to negative portrayals of men in the drama, which could explain their results at endline as compared with men with consistent exposure.

Evidence of three pathways of change emerged from the qualitative data. The first was that participants learned key messages alongside characters, including caregivers who could then help maintain key messages by repeating them to their daughters after the intervention concluded. Secondly, we found that character's role modelled gender equitable behaviors by providing lived examples, and concrete, relatable consequences of both positive and negative behaviors. Lastly, the drama provided language and context that facilitated conversations about age-disparate transactional sex between caregivers and their daughters, fostering critical reflection about how to support adolescent girls.

### 4.2. Strengths and limitations

This study had several strengths and limitations. Our sample may have been biased towards those with a higher level of education as only participant's comfortable speaking Kiswahili (the language of the radio drama, which is taught in schools) were included. Longitudinal qualitative data from adolescent girls, and women and men caregivers, however, provided rich evidence into perceptions and experiences of the intervention from a wide range of perspectives, suggesting our findings may be generalizable and transferable. Secondly, none of the participants reported engaging in age-disparate transactional sex despite stating it was prevalent in their communities. This may have been partially due to social desirability bias, especially among adolescent girls given the sensitivity of the topic. As our primary aim was to change social norms rather than behaviors, however, this was not a major limitation.

Given the small quantitative sample our regression analyses were exploratory, and the results presented preliminary. We were not able to run analyses by disability status as our sample of participants living with a disability was too small (*n* = 7), nor estimate any random variance parameters to account for the nesting of participants within 24 villages. The chance of Type I error (or “false positive”) was not low given the small sample and the number of models run, but outcomes were consistent across models.

It is an ongoing challenge in implementation research to determine what constitutes meaningful intervention exposure (40), and our study is not unique in having possibly violated this consistency assumption (41). In particular, our intention-to-treat results should be interpreted cautiously as there were challenges implementing discussion sessions. Additionally, our per-protocol analyses relied on self-reported exposure which could have resulted in under or over estimation of its impact (42). In response, we tested the impacts of different levels of intervention exposure. The pre-post design and presence of few confounders of the exposure-outcome relationship allow our results to be broadly interpretable as overall change attributable to the intervention, and our mixed-methods data suggest important indications of change resulting from the LINEA intervention; although we cannot determine causation because of the lack of control group in our design.

### 4.3. Implications

Although evidence from this study must be interpreted with caution, our findings have important implications for research and programs. Overall, we found robust qualitative evidence of change at the individual level of knowledge, behaviours, beliefs and attitudes, suggesting “edutainment” interventions may be effective as stand-alone programmes, even in the short-term. This is contrary to findings from a recent literature review (43), and more research is needed to test it. We found less evidence of change at the communal level, which is in line with the literature that norms are harder to shift than beliefs and require longer intervention periods (15).

Our findings also suggest it's possible to inadvertently reinforce gender inequitable norms, especially amongst men, if they do not fully adhere to the intervention. The challenges of engaging men in gender-transformative interventions to prevent violence against women and girls have been well documented; men may not see these interventions as relevant to them or may perceive them as a threat to their own masculinities (26) and an infringement on their rights (27). Past evaluations have also found men often don't maintain gender equitable behaviors consistently or in the long-term (44), especially the most gender inequitable men and those who perpetrate gender-based violence (45). This highlights the importance of encouraging, or possibly incentivizing men to listen to the radio drama until the end, exploring different times of day and radio stations the drama could be broadcast on, and further engaging them through the curricula sessions intended in the full-scale intervention. Additional factors that have been suggested to help shift deeply entrenched gender inequitable norms among men include male role models who hold them accountable (46), including women in interventions, and acknowledging and intervening on structural factors disempowering men (27, 47), all of which could be capitalized in future program development and implementation.

## 5. Conclusion

Our findings support piloting of the LINEA intervention in other regions of Tanzania. Our data has shown that even after a reduced implementation period (seven weeks), high engagement with the intervention leads to desirable changes in knowledge, behaviors, attitudes and beliefs driving age-disparate transactional sex, especially among adolescent girls. These findings were largely driven by our qualitative results, which also indicated potential future social norms change, and elucidated pathways through which the intervention effects change.

Our quantitative analyses indicate gender inequitable norms may be marginally reinforced amongst adults, and especially men, if they are not adequately engaged in the intervention. This suggests they could benefit from the nine-months of implementation and participation in the curricula sessions intended in the full-scale intervention, and additional critical engagement and encouragement to listen to the radio drama until the end.

Overall, these indications of change are very promising and suggest when implemented as intended the LINEA intervention could lead to sustained, gender-transformative change. The attitudes, beliefs and social norms underpinning age-disparate transactional sex also underpin sexual violence against adolescent girls and gender-based violence more generally, suggesting the intervention may have wider-reaching impacts. If proven effective through a robust impact evaluation, this mass-media intervention has the potential to be scaled-up at the national level, including in different geographic regions and hard-to-reach communities (23).

## Data Availability

The raw data supporting the conclusions of this article will be made available by the authors, without undue reservation.

## Footnotes

^1^ www.lshtm.ac.uk/linea
