# Supplementary material for: A mixed-methods, exploratory, quasi-experimental evaluation of a radio drama intervention to prevent age-disparate transactional sex in Tanzania
Source: Front Reprod Health. 2022 Dec 2;4:1000853. doi: 10.3389/frph.2022.1000853 (PMC9755860; doi:10.3389/frph.2022.1000853)
Supplement: Supplementary file 1 [file Datasheet1.docx]

Supplementary Material 1


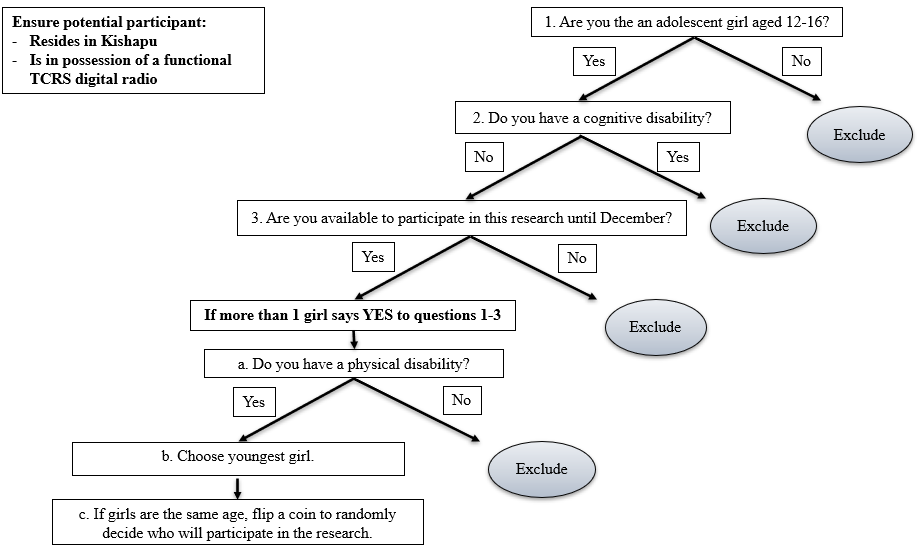


**SUPPLEMENTARY FIGURE 1**

Sampling decision tree for adolescent girls in English


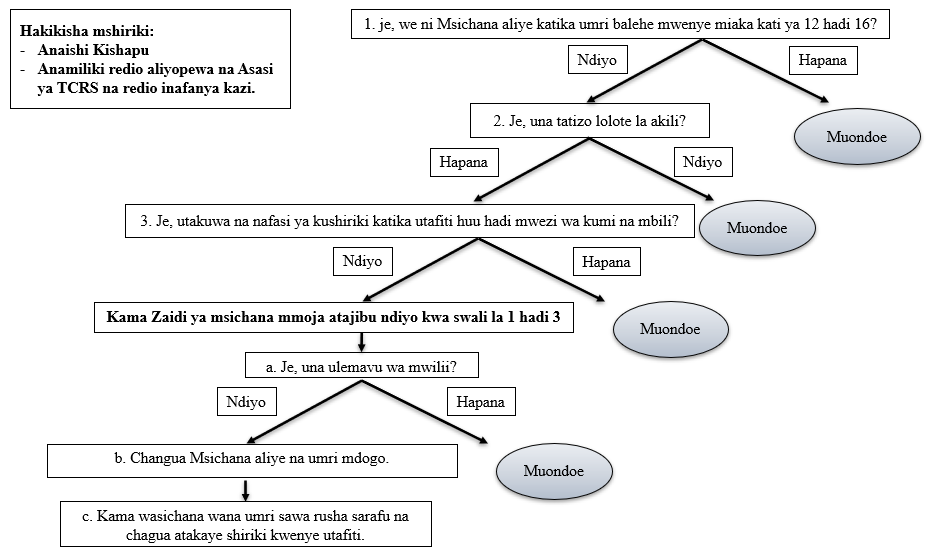


**SUPPLEMENTARY FIGURE 2**

Sampling decision tree for adolescent girls in Kiswahili


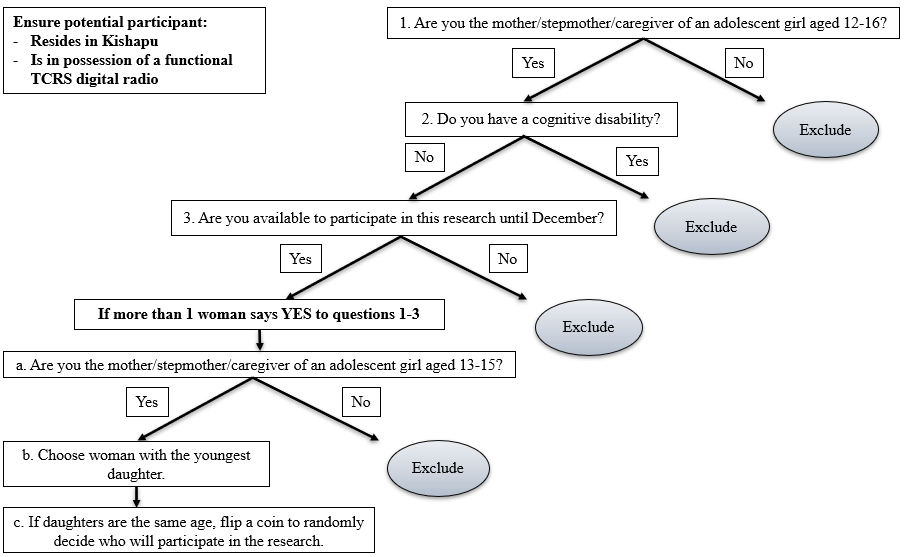


**SUPPLEMENTARY FIGURE 3**

Sampling decision tree for women caregivers in English


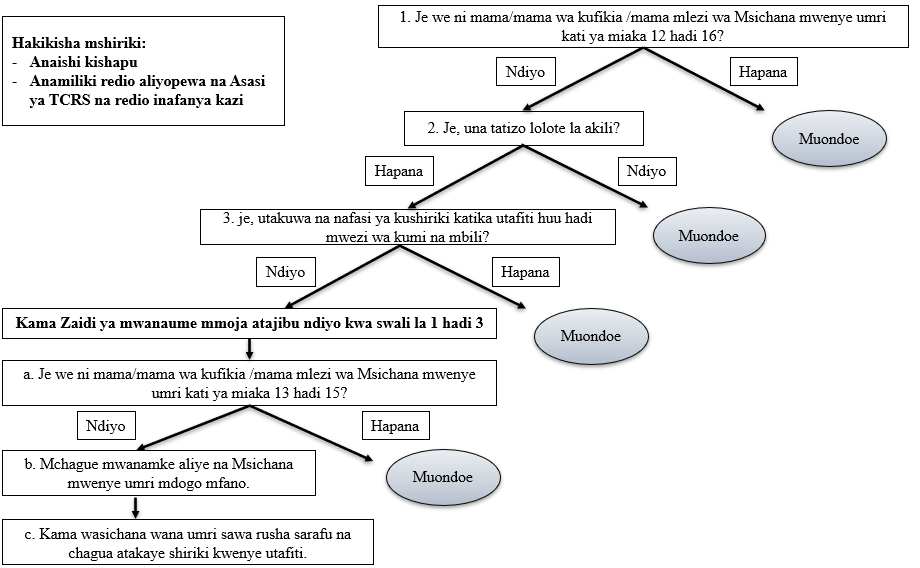


**SUPPLEMENTARY FIGURE 4**

Sampling decision tree for women caregivers in Kiswahili


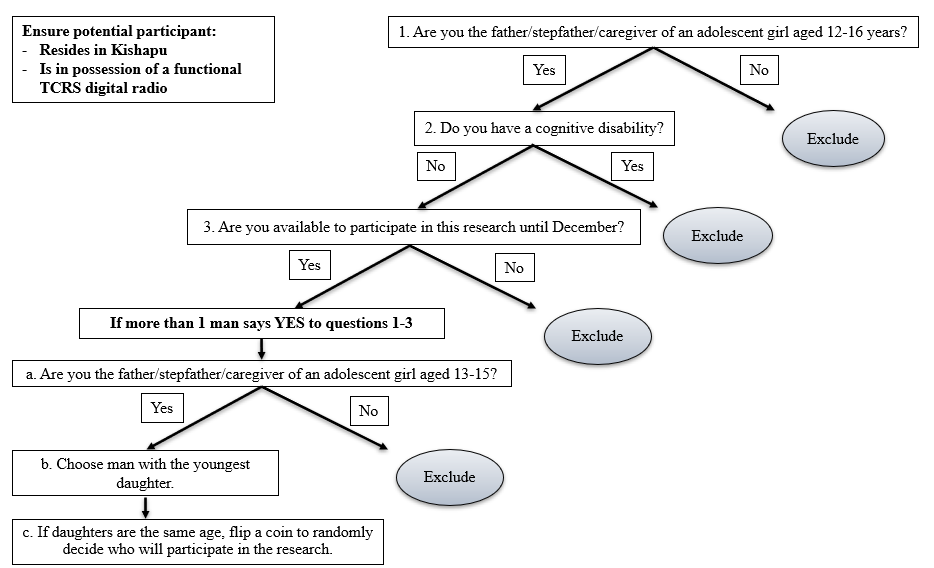


**Supplementary Figure 5**

Sampling decision tree for men caregivers in English


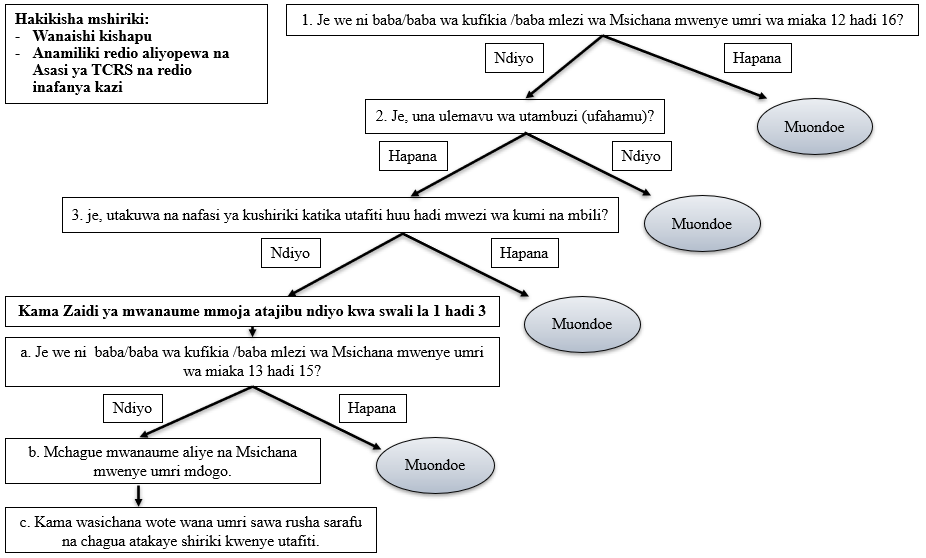


**SUPPLEMENTARY FIGURE 6**

Sampling decision tree for men caregivers in Kiswahili
