# Supplementary material for: A mixed-methods, exploratory, quasi-experimental evaluation of a radio drama intervention to prevent age-disparate transactional sex in Tanzania
Source: Front Reprod Health. 2022 Dec 2;4:1000853. doi: 10.3389/frph.2022.1000853 (PMC9755860; doi:10.3389/frph.2022.1000853)
Supplement: Supplementary file 2 [file Datasheet2.docx]

Supplementary Material 2

# The Norms and Attitudes on Age Disparate Transactional Sex Scale (NAATSS) items in English

For this part of the interview, I will read you a statement and then ask you how much you agree or disagree with the statement.  For each statement I want you to tell me if you 1 strongly disagree, 2 disagree, 3 agree, or 4 strongly agree.

| 1 | 2 | 3 | 4 |
| --- | --- | --- | --- |
| Strongly Disagree | Disagree | Agree | Strongly Agree |

| **NAATSS** | **Attributions to Girls’ Behavior** |
| --- | --- |
|  | In the opinion of residents of your community, if a girl responds positively to the advances of a man on the street, she should have sex with him. |
|  | In the opinion of residents of your community, girls that accept presents from men should repay with sex. |
|  | In the opinion of residents of your community, girls that use short skirts are looking for male attention. |
|  | In the opinion of residents of your community, girls that spend a lot of time in the street are available to get involved with men. |
|  | In the opinion of residents of your community, girls get involved with men to gain financial stability. |
|  | **Men’s Motivations** |
| 6. | In the opinion of residents of your community, men feel more powerful in sexual relations with girls than with women. |
| 7. | In the opinion of residents of your community, men get more pleasure from sex with girls than with women. |
|  | In the opinion of residents of your community, men think that the body of a girl is more attractive than that of a woman. |
|  | In the opinion of residents of your community, men like to get involved with girls because they are easier to control than women. |
|  | In the opinion of residents of your community, men never refuse an opportunity to have sex with a girl. |
|  | **Girls’ Readiness to have Sex** |
|  | In the opinion of residents of your community, a girl with a developed body has the maturity to make decisions about her sexual partners and relations. |
|  | In the opinion of residents of your community, a girl with a developed body is ready to have sex. |
|  | In the opinion of residents of your community, girls of 12 to 16 years old are able to choose their sexual partners and relationships. |

# The Norms and Attitudes on Age Disparate Transactional Sex Scale (NAATSS) items in Kiswahili

Kwa sehemu hii ya mahojiano,nitasoma sentensi na nitakuuliza ni kwa kiwango gani unakubaliana nayo au haukubaliani nayo. Kwa kila sentensi nataka uniambie kama 1 Sikubaliani kabisa, 2 Sikubaliani, 3 Nakubali, 4 Nakubaliana kabisa.

| 1 | 2 | 3 | 4 |
| --- | --- | --- | --- |
| Sikubaliani kabisa | Sikubaliani | Nakubali | Nakubaliana kabisa |

| **NAATSS** | **Sifa za tabia za wasichana** |
| --- | --- |
|  | Kwa maoni ya wakazi wa jamii yako, ikiwa msichana ataonesha mwitikio chanya juu ya ushawishi wa mwanaume mtaani, Hii inamaanisha yuko tayari kufanya naye ngono. |
|  | Kwa maoni ya wakazi wa jamii yako, wasichana wanaokubali zawadi kutoka kwa wanaume, wanapaswa kulipa kwa kufanya nao ngono. |
|  | Kwa maoni ya wakazi wa jamii yako, wasichana wanaovaa sketi fupi, hufanya hivyo ili kushawishi wanaume. |
|  | Kwa maoni ya wakazi wa jamii yako, wasichana ambao hutumia muda mwingi mitaani, mara nyingi huwa wako na wanaume. |
|  | Kwa maoni ya wakazi wa jamii yako, wasichana huanzisha uhusiano na wanaume ili kupata fedha. |
|  | **Motisha za wanaume** |
|  | Kwa maoni ya wakazi wa jamii yako, wanaume huhisi wana uwezo zaidi kwa kuwa na uhusiano wa kimapenzi na wasichana wadogo kuliko na wanawake watu wazima. |
|  | Kwa maoni ya wakazi wa jamii yako, wanaume hufurahia zaidi kufanya ngono na wasichana wadogo kuliko na wanawake watu wazima. |
|  | Kwa maoni ya wakazi wa jamii yako, wanaume hufikiria kuwa mwili wa msichana mdogo unavutia zaidi kuliko ule wa mwanamke. |
|  | Kwa maoni ya wakazi wa jamii yako, wanaume hupendelea zaidi kuwa kwenye mahusiano na wasichana wadogo kwa sababu ni rahisi kuwatawala kuliko wanawake. |
|  | Kwa maoni ya wakazi wa jamii yako, wanaume hawakatai fursa/nafasi ya kufanya ngono na msichana. |
|  | **Utayari wa wasichana kufanya ngono** |
|  | Kwa maoni ya wakazi wa jamii yako, msichana aliyekomaa kimwili ana maamuzi ya kuingia kwenye mahusiano na kufanya ngono. |
|  | Kwa maoni ya wakazi wa jamii yako, msichana aliyekomaa kimwili yuko tayari kufanya ngono. |
|  | Kwa maoni ya wakazi wa jamii yako, wasichana wa miaka 12 hadi 16 wana uwezo wa kuchagua wapenzi wao wa kingono na mahusiano. |
